# A Practical Treatise on the Diseases and Injuries of the Urinary Bladder, the Prostate Gland, and the Urethra

**Published:** 1851-09

**Authors:** 


### REVIEWS.

Art. VI.—*A Practical Treatise on the Diseases and Injuries of the Urinary Bladder, the Prostate Gland, and the Urethra. By* S. D. Gross, M. D., Professor of Surgery in the University of Louisville, Member of the American Medical Association; author of Elements of Pathological Anatomy, etc. etc. With one hundred and six illustrations. Philadelphia: Blanchard & Lea. 1851. pp. 726. 8vo.

The bibliography of the diseases of the urinary organs, until the publication of the volume whose title stands at the head of this article, has been singularly and deplorably defective. When we consider the importance, both in a practical and pathological point of view, of the affections which have their seat in the urinary organs, this defect becomes a matter of much surprise. Fewer attempts have been made to compose systematic treatises on this subject than almost any other—certainly than any other of equal importance—in the whole range of medicine.

Soemmering, a German, who flourished during the last century, left a small volume of one hundred and forty or fifty pages on these diseases which remains, to this day, the only attempt at a systematic treatise on the subject in the German language. This work, besides being far too small to embrace one-half the diseases to which these parts are subject, is chiefly a reproduction from the French, or, to speak of it in the mildest terms, is based upon French labors.

M. Civiale published, in 1837, the first volume of his work on "Diseases of the Genito-Urinary Organs." This was followed, four years after, by volume the second, and this, in another year, by volume the third and last. The treatise of Civiale, while most admirable in some respects, is inexcusably deficient in others, and is, on the whole, we think, far inferior to some other and less pretending works published on the other side of the Channel. Good or bad, however, it is the best, and only original French work on the diseases in question. Never having been translated into English, it is inaccessible to the majority of American readers.

Willis, Coulson, and Sir Benjamin Brodie, are the three English authors who have written most extensively on this subject. Mr. Guthrie, of the Westminster Hospital, published, some years ago, the first volume of a work entitled the "Anatomy and Diseases of the Urinary and Sexual Organs," with the promise to follow it without much delay by a second part which was to contain those subjects—and they were many and important—omitted in the first. It is to be regretted that this distinguished surgeon has not yet fulfilled his promise, and it is to be hoped that he will not allow many years to elapse before doing so.

The work of Dr. Willis, of London, was rather more on the Urine than on the diseases of the Urinary Organs.

Coulson wrote a short treatise which is anything but systematic, and much behind the present state of the science.

The author to whom English readers have been most indebted for a description of these diseases and their treatment, is Sir Benjamin Brodie. This distinguished surgeon has made most valuable contributions on these subjects in his usual clear, forcible style. But, taken as a whole, his work on "Diseases of the Urinary Organs" does not meet the wants of the profession, especially in this country.

A monograph which should contain a full and comprehensive exposition of the nature, causes, symptoms, and treatment of the diseases in question has, indeed, long been an acknowledged desideratum with the profession, and has now been supplied by the treatise of Dr. Gross.

The introduction to this work, which occupies fifty or sixty pages, is devoted to a description of the anatomy of the perineum, of the urinary bladder, of the prostate and urethra, and to an account of the urine, with its different varieties, sediments, changes in disease, &c.

In addition to the description usually contained in anatomical works, of the *superficial fascia* of the perineum, Dr. Gross has embodied the very important discoveries recently made by Dr. Buck, one of the surgeons to the New York Hospital. Dr. Buck has demonstrated by careful dissections, one of which Dr. Gross had an opportunity of witnessing, that the perineal fascia forms distinct sheaths for the perineal muscles, the spongy structure of the urethra, and the cavernous bodies of the penis. This fascia answers the same purpose for these bodies, this structure, and these muscles, that the fascia lata does for the muscles of the thigh. Forming a complete sheath to these parts, which serves to maintain them in their respective places, it also separates them from each other, and thereby limits their diseases. The arrangement, relations and connections of this membrane render it evident, that urine effused in consequence of a rupture of the urethra, can travel only in one direction, namely, forward towards the scrotum, and upward towards the groin. The attachment of the membrane to the pelvic bones and the triangular ligament forbids its passage backwards round the anus and along the inside of the thighs. It occasionally happens, however, that the fluid breaks through this barrier and makes its way along the inside of the lower extremities. In one case, it is reported to have reached nearly as far down as the knee. But we cannot dwell longer on this portion of the work.

The bladder receives a due share of notice. The prostate is described at length, and with great minuteness, and its anatomy is illustrated by a number of accurate and well executed cuts.

The urethra is divided into the usual portions, and is also illustrated by plates. In speaking of the first, or prostatic division of the urethra, Dr. Gross states that in most of his examinations, he has found "the urethra situated towards the superior part of the prostate, in a sort of gutter, covered merely by a thin layer of parenchymatous substance. Sometimes the tube runs through the centre of the gland, which thus forms a kind of hollow cylinder for it. Occasionally, again, though very rarely, the urethra occupies the inferior part of this organ, the greater volume, by far, lying upon its upper surface. When this arrangement obtains, the canal and the rectum approach each other much nearer than usual, in consequence of which the latter is in danger of being wounded in the operation for stone." Fortunately, however, this condition of the parts is, as has been stated, exceedingly rare; otherwise the bowel would more frequently be the subject of injury in this operation. In the adult, the direction of the prostatic portion of the urethra, in the erect position of the body, is obliquely downwards and forwards; but in the aged subject it is drawn down by the bas-fond of the bladder, and is, consequently, more horizontal. In childhood, owing to the fact that the bladder is in a great degree lodged in the abdomen, the prostatic portion of the urethra is nearly vertical. These facts are both interesting and important to the lithotomist.

The fourth and last chapter of the introduction is devoted to the Urine. The characters of this fluid in the normal state of the system, and the many changes which it undergoes in the various disorders of the body, are stated succinctly; but having been so often described we will not here detain the reader with an account of them.

We arrive now at "*Diseases of the Urinary Organs.*"

And first of diseases and injuries of the bladder. The author, very properly, gives a brief account of the *malformations* and *imperfections* of this viscus. Although the malformations of the bladder are seldom met with, and in a practical point of view, are not of much importance, they are sufficiently interesting to merit a passing notice. They are arranged under three heads: 1. Absence of the bladder: 2. Bilobation, or multiplication of the organ: 3. Extrophy, or congenital eversion.

The first defect is rare, and when observed, the ureters open at one of three places: Either into the vagina, the rectum, or the urethra. Haller makes mention of cases in which the ureters were inserted into the vagina. Richardson, in the Transactions of the Philosophical Society of London, has published the history of a youth who attained the age of seventeen years without ever having urinated by the penis. He passed all his water by the anus.

There are few cases on record in which the ureters open directly into the urethra. The body of Abraham Clef affords perhaps the best authenticated example of this *lusus naturae*.

The effect of disease has sometimes been to divide the bladder into two or more compartments. This defect has sometimes also been congenital. The most remarkable example of congenital malformation occurs in the writings of Molinetti. The individual, a female, had five bladders, five kidneys, and six ureters, of which two were inserted into the largest reservoir, while the remainder terminated each in one of the small sacs, which discharged their contents by special ducts into the main organs.

In a patient who had been subject to retention of urine, the bladder was found divided into two nearly equal sacs, the one situated behind the other, and communicating together by an openining of some size.

Our author very justly, we think, mistrusts most of the cases of supernumerary bladders mentioned by the older anatomists.

Congenital inversion or extrophy, which "consists in the absence of the anterior wall of the bladder, and the protrusion of its posterior wall across an opening in the inferior part of the linea abba," is the most remarkable malformation of this organ. Happily it is the most rare. Dr. Gross has seen but one example of this monstrosity, a description of which he gives in the following words:—

"Joseph Head, nineteen years old, a native of Rhode Island, is five feet six inches high and well formed, but has never enjoyed robust health having had ten different attacks of bilious fever. His intelligence is very ordinary, and he is a painter by trade, but has never worked much at his business. He has a brother and a sister who are both sound and well formed. Walking is painful to him, from the pressure and friction of his pantaloons upon the tumor; he constanily keeps the part covered with a thick, soft compre s, to imb be the urine, changing it five or six times a day. His bowels are habitually constipated. His body and limbs are well proportioned and well developed, but he has no beard.

"The bladder is situated just above the pubic symphysis, in the median line, where it forms a rounded, convex tumor, four inches in breadth by three inches and a quarter in height. The tumor is soft and elastic, has a red, raw appearance, bleeds easily when touched, and is tender and even painful on pressure. Its size is increased when the patient stands up, and diminished when he lies down. The upper, central part of the tumor is covered with skin, a sort of imperfect copy of the original; everywhere else, it is red and raw. The skin, at its periphery, especially on the left side, is somewhat puckered, and has the aspect of a cicatrice. The orifices of the ureters, indicated by a frequent oozing, or occasional jet of urine, are quite small, and one inch and three-eighths of an inch apart: the right is mammillated, and more prominent than the left. Between the tumor and the stunted penis a deep furrow exists, at the bottom of which are the mouths of the ejaculatory ducts, separated by an interval five-eighths of an inch in width.

"The interval between the pubic bones is four inches, and appears to be occupied by a dense, elastic structure, probably fibro-ligamentous in its character. The bodies and symphysis of these bones are evidently wanting.

"The penis is imperforate, and consists of two rudimentary cavernous bodies. In its flacid condition, it is upwards of two inches in its transverse diameter, and ten lines in the antero-posterior; it has a prepuce, and a tolerably distinct frænum in the usual situation. The organ is flattened above, where it is in contact with the tumor. During erection, it increases to nearly double the ordinary size; the act, however, is unattended by any pleasurable sensation; on the contrary, it is always more or less painful, from the pressure which the distended organ exerts upon the tumor. A mucilaginous fluid, probably prostatic liquor, passes off involuntarily every day, and once or twice a week there are considerable seminal emissions, without any very agreeable feeling or emotion. The patient has at no time any amorous propensities.

"The scrotum is smaller than usual, and its surface is covered by a few straggling hairs. The spermatic vessels and ducts appear to be of the natural size and consistence. The testicles are well formed: the left hangs lower than the right. There is hernia on the right side, but the bowel does not descend into the scrotum.

"No umbilicus is perceptible; one must, undoubtedly, have existed, but at what point cannot now be determined. Possibly, the umbilical vessels, which naturally run along the sides of the bladder, in their course towards the linea alba, may have issued at the place of union of the abdominal and vesical parietes, and thus prevented the formation of a navel, properly so called.

"The groins are pretty thickly covered with hair, which extends unusually far outwards towards the spine of the ilium; it is of the ordinary length, and of the same color as that of the head."

Extrophy of the bladder is wholly incurable.

*Wounds of the bladder* are dismissed by most authors without ceremony, and yet they are by no means infrequent, and are invested with very great practical and pathological interest. The form of wounds of the bladder depends upon the kind of instrument with which they are inflicted. Thus they may be lacerated, punctured, incised, or gun-shot. In whatever manner inflicted, a wound may transfix the bladder or merely pierce one of its walls; two openings would result in the former case; in the latter, only one. The lesion may occur in front and below, where the organ is destitute of peritoneum, or at a point where covered by this investment.

The incision made in recto-vesical and supra-pubic operations, affords illustrations of *incised* wounds of the bladder. The trochar when introduced in cases where obstruction of the urethra makes it necessary to draw off the urine, furnishes an example of a punctured wound.

A few centuries ago all wounds of the bladder, however small and insignificant, were considered necessarily fatal within a short time after their occurrence. This, however, is far from being true. The fatality of these accidents depends very much upon the amount of the accompanying violence, the point, size, direction and number of the opening, the quantity of urine contained in the bladder at the time, &c. The opening being small, penetrating the cavity of the bladder obliquely, the viscus being at the time nearly or quite empty, escape of urine may be prevented, and the wound healed by the first intention. A wound affecting the body or fundus of the organ, inflicted upon a distended bladder, is more hazardous than one inflicted upon an empty bladder, and at its inferior part. Incised and punctured wounds are far more dangerous than gun-shot wounds and nearly always prove fatal. Mr. Colles states that gun-shot wounds of the bladder are not always mortal, and refers to a case in which a man recovered after having received a ball that went through the sciatic notch, and penetrated the cavity of the bladder, where it lodged, and remained for twelve months, when it was removed. Writers mention very many cases in which the bladder has been penetrated by musket balls and the individuals have recovered.

The first effort of the surgeon, when called to treat a wounded bladder, is to prevent extravasation of urine; and secondly, the development of undue inflammation.

"Unfortunately the first of these accidents often takes place at the moment of the injury, and consequently before the surgeon has an opportunity of interfering. When the bladder is distended, it matters not where it is laid open, whether at a part invested by peritoneum or not, effusion of urine will be inevitable; the danger of the case will thus be increased, in an instant, an hundred fold. When the general cavity of the abdomen is penetrated, the contact of the fluid will in a few hours set up intense peritonitis, which no skill can possibly control. The disease proceeds in spite of the best directed efforts to combat it. This being the fact, the patient's only chance consists in preventing its occurrence. This is to be attempted by attention to position, and by the instant evacuation of the bladder. The patient should be placed almost semi-erect in bed, and the catheter, which should be of gumelastic, should be left in the bladder, where it is to be secured in the usual manner, to enable the urine to pass off as fast as it comes down from the ureters. In a word, the organ should be kept constantly empty and contracted for the first fifteen or twenty days, or until there is reason to conclude that the wound is closed, and all risk of infiltration over. The end of the instrument must not be permitted to become clogged, or to rise up in the bladder, otherwise the object for which it is employed will not be attained. Care should also be taken that it do not press or rub against the mucous membrane, and thereby excite pain, spasm, or irritation, rendering its presence uncomfortable, if not intolerable. Should the latter result, however, follow, the catheter must be withdrawn, and an attempt made to obviate the danger of distension by the frequent introduction of the instrument.

"The development of undue inflammation is to be prevented by the employment of antiphlogistic means. Foremost amongst these are general and local bleeding, calomel and opium, hot fomentations, and vesication of the abdomen. Anodynes must be given in full doses, both by the mouth and by the rectum, to allay pain and spasm of the bladder, induce sleep, and diminish the renal secretion. The drinks must be cooling and demulcent, the diet perfectly light and bland, and the bowels must be disturbed as little as possible during the first eight or ten days. No drastic purgatives are admissible. The best aperients are castor oil and sulphate of magnesia. Cathartic enemata must be avoided, on account of the pain and irritation which they produce by their pressure on the bladder. Abscesses, the result of urinous infiltration, are to be opened by early and free incisions.

"Nothing can be gained by an attempt to extract the foreign body, when the injury has been produced by firearms; for the very moment it is inflicted the urine escapes, and the bladder contracts upon itself so as to destroy the relations between the external and internal wounds. If the ball has fallen into the bladder, it may, if not too large, either pass off spontaneously, or be removed with the forceps; should it be otherwise, and severe symptoms be caused by its presence, it must be cut out through the perinæum by an operation similar to that of lithotomy. This may be done immediately or within a short period after the accident, if the ball has entered beneath the pubes, for the reason that the organ will not only be freed thereby of a disagreeable intruder, but also because there will be less risk of urinous infiltration.

"When the bladder has been transfixed, or wounded through the peritoneum, the accident, as all experience proves, inevitably terminates fatally. In view of this event, would it be proper to make an incision through the linea alba, and sponge out the extravasated fluid? My opinion is that it would, and that it would be much more creditable to a surgeon to perform such an operation, provided it can be done immediately after the injury has been received, than to stand by, and see his patient perish from the effects of peritonitis. The only difficulty in the case might be the uncertainty of the abdominal effusion."

*Laceration* of the bladder is the subject next discussed. Although this accident is generally immediately fatal, our author has thought it of sufficient importance to describe its causes, syptoms, and treatment, which he has done with much accuracy.

*Cystitis*, in the acute form, is seldom met with; Dr. Gross has seen comparatively few cases of the complaint. Chronic cystitis is, on the contrary, by no means infrequent, and gives rise to much suffering, which often continues for months and years, and finally destroys life. Increased vascularity, deposits of lymph, loss of transparency, softening, and alteration of the natural secretion, are the more important anatomical characters of this affection. Calculous diseases, traumatic lesions of the bladder, stricture of the urethra, retention of urine, enlargement of the prostate gland, and violent exercise on horseback, are among the many and most frequent causes of acute inflammation of the bladder.

The *symptoms* of cystitis are sufficiently well known to be omitted in this place. The particular seat of the morbid action, however, affords our author an opportunity of speaking of its differential diagnosis, in which, we may remark, he has excelled all preceding writers on the subject. When the inflammation is situated at the bas-fond, the rectum usually sympathizes, and there is straining and tenesmus. When the neck of the bladder is the seat of the disease, there is obstinate retention of the urine, accompanied by excessive pain, and a sense of weight in the anus and perineum, with a constant desire to urinate, and severe burning from one extremity of the urethra to the other. The desire to micturate is less frequent, and the pain about the neck of the bladder less severe, when the inflammation attacks the anterior wall of the bladder. There is also, in addition, a feeling of constriction in the hypogastric region, with tenderness on pressure and percussion; the patient generally lies on his side, with the knees partially flexed, in order to relieve the tension of the abdominal walls.

The progress and termination are greatly modified both by its cause and seat. The traumatic form of the disease is more formidable than the idiopathic; the complication with enlargement of the prostate gland, or organic change of the kidneys, or stricture of the urethra, militates greatly against recovery.

The treatment of cystitis does not differ sufficiently from that required in inflammation of other parts of the body, to require a special description.

As an idiopathic affection, *fibrinous exudation* of the bladder is seldom met with; as the consequence of external injury, or direct irritation of the mucous membrane, it is not uncommon. The extraction of a stone is sometimes rendered very difficult—as we ourselves, on the occasion of our first operation of lithotomy, experienced—by this exudation attaching the calculus to the walls of the bladder. It is through the agency of fibrin that the various foreign bodies, which occasionally find their way into the bladder, are encysted. When this substance is not discharged with the same rapidity with which it is secreted, it occasionally gives rise to retention of urine, by blocking up the urethra, and when it extends upwards into the ureters, it dams up the urine in the kidneys.

The presence of some of the exuded matter in the urine, is the only symptom, and this, certainly, is far from being a sufficient one, to enable us to distinguish this form of inflammation from ordinary cystitis. Internal remedies appear to exert no controlling influence over this secretion. Injections with nitrate of silver may prevent its effusion, which, if its existence in very great quantities can be positively ascertained, should be evacuated by the "high operation."

*Suppuration*, *Abscess*, *Gangrene and Ulceration of the Bladder*, are discussed in the remaining sections of this chapter. These are affections which are seldom encountered, and are in almost all cases incurable. Ulceration is amongst the very rarest diseases to which this organ is subject. The structure, situation, and functions of the bladder, and nature of the urine are all opposed in an eminent degree to the reparation of ulcers of this viscus. And, yet, this process sometimes takes place; that it is very rare, is attested by daily experience. Those who believe that ulcers of this organ do occasionally heal, support their opinion first, by cases of this disease in which the ulcers have been found incrusted with coagulable lymph; and secondly, by instances in which the mucous membrane has exhibited a puckered and contracted appearance, strongly indicative of complete cicatrization. Finally, analogy, or what is continually observed to occur in other portions of the mucous system—as in the alimentary canal, for instance, or the mouth, or vagina, or uterus—affords very strong, if not conclusive proof of the possibility of this occurrence. Still, as we have remarked, it is very infrequent.

The usual seat of ulceration is the bas-fond and neck of the bladder. Mechanical causes such as calculous concretions, or sandy deposits and tubercular disease; organic lesion of the kidney, paralysis of the bladder, injury of the spinal cord—which three last induce changes in the composition of the urine—cystitis, dependent on enlargement of the prostate, and stricture of the urethra, and chronic inflammation of long standing, are the conditions to which ulceration of the bladder is most frequently to be traced.

At the commencement of the disease, the symptoms of ulcerated bladder are not materially different from those observed in subacute, or chronic inflammation. Nor indeed are they by any means always characteristic at a later period. Such, in fact, is the obscurity which envelops this disease, that it is often only by the aid of the scalpel that we are enabled to determine its existence.

Frequently the most experieneed surgeons have been entirely baffled in its diagnosis. Stone, cystitis, and catarh—diseases on which, as we have remarked, it often depends—are the affections with which it is most liable to be confounded.

"In stone, the pain is most severe immediately after passing the urine, and is generally much aggravated by rough exercise, the urine is also more frequently bloody, there is less irritability of the urethra, and the intervals between the paroxysms are longer than in ulceration."

The guarded and gentle use of the sound—in order to avoid an aggravation of the local inflammation if the case be one of ulceration,—will greatly facilitate the diagnosis.

It will be distinguished from cystitis "by its obstinate persistence, by the greater extent and violence of the local distress, by the incessant desire to void the urine, which is never suffered to accumulate, by the more severe burnings or scaldings along the urethra, and, lastly, by the presence of pus in the urine, and in the more aggravated forms of the complaint, the absence of mucus."

The characteristic symptom of catarrh, the turbid urine with its ammoniacal odor, the presence of much less local and constitutional distress, the complete intermission of the vesical disturbance, added to the fact that the calls to make water are not so frequent, and the sensibility of the urethra not so great, will usually enable the practitioner to distinguish this affection from ulceration of the bladder.

A symptom which is never furnished by either catarrh, simple cystitis, or calculous disease, is a discharge of the debris of the mucous membrane—a circumstance occasionally observed during the progress of ulceration.

The *treatment*—which is general and local—is conducted upon well known principles and is admirably set forth under the head of cystitis.

Dr. Gross closes this portion of his work with the detail of three cases of ulceration of the bladder copied from different sources. Since their publication his practice has afforded an interesting example of this affection which had its origin in tubercular disease. We had intended incorporating a description of this case in the present article, but as Dr. Gross purposes giving an account of it in an early number of this Journal, we leave it to his abler hands.

*Chronic lesions of the Bladder* are next introduced to our notice. *Catarrh and Hypertrophy* are treated of in section first.

Cystorrhea is generally a symptom merely of a more serious disease, and consists of an excessive secretion of white, glairy mucus. Our author discards the division of cystorrhea into the acute and chronic varieties, and supports this step by referring to the fact—and it is one—that the former affection does not differ in any respect from ordinary acute cystitis.

Cystorrhea is a disease almost peculiar to advanced life, and is very seldom seen before the forty-fifth or fiftieth year. Dr. Gross has never met with it before puberty except as an attendant upon stone.

The causes of cystorrhea are in many respects identical with those of cystitis and ulceration of the bladder. Once established, it is readily reproduced or aggravated by any of the usual causes of inflammation, and particularly of inflammation of the bladder.

The diagnosis of this disease is never difficult. "Its characteristic symptom is an inordinate discharge of mucus, dependent upon chronic or subacute inflammation of the lining membrane, and accompanied with frequent, painful, and difficult micturition. Almost the only affection with which it is liable to be confounded is seminal emission; but this can only happen when the seminal fluid flows into the bladder, and mixes with the urine, in consequence of a stricture of the urethra, or enlargement of the prostate gland. The distinction is, that in catarrh the discharge is always greater, more constant, and also more ropy, tenacious, and offensive; the local suffering is always more severe, and there is a more frequent desire to urinate. In seminal disease, the matter is voided in small quantity, and at remote intervals; it has a peculiar odor, is of light color, and is insoluble in the water, in which it floats about in shreds. When there is any doubt, the best way is to submit a few drops of the suspected fluid to microscopical examination. If it be semen, it will be found to consist of small oblong bodies, with delicate tapering tails. From gonorrhea it is readily distinguished by the character of the discharge, the absence of urethritis, and the history of the case. Its connexion with stone can only be determined by sounding. In suppuration of the bladder, the discharge is of a yellowish color, globular in its character, and specifically heavier than in catarrh."

In the treatment of no disease of the bladder is it more essential than in catarrh, to determine the nature of the exciting cause; for in none are the remedies more various or more opposite in their qualities and effects. An acquaintance with the cause at once opens up in this, as in the analogous diseases, the proper plan to be pursued. In fact, the probabilities of cure in nearly all cases are governed by the period at which we remove the exciting cause, especially when this cause offers an impediment to the free discharge of the urine.

Antiphlogistics, in the form of the general and local abstraction of blood, and purgatives—carefully abstaining from those which tend to irritate the rectum,—the warm bath, warm enemata, warm fomentations, demulcent drinks, &c., are required in all cases attended with violent pain and frequent micturition, though there may be no constitutional disturbance. With these remedies we will sometimes succeed in subjugating the more distressing symptoms in the course of a very short time. After this has been done, the remedy which is regarded by Dr. Gross as affording the greatest relief to the patient, is the balsam of copaiba, administered three or four times in the twenty-four hours, in doses not exceeding ten, fifteen or twenty drops. When exhibited in large quantities, instead of ameliorating the condition of things, it serves to increase the urinary irritation.

Our author thinks well of the terebinthinate preparations—of which he esteems the Chian turpentine the best—used in small doses, in combination with extract of henbane, &c. From the *pareira brava* and *buchu*—both articles highly extolled in the treatment of cystorrhea—he has never derived much advantage. The former gives rise to nausea and other disagreeable effects, without appearing to exert any special influence upon the urinary apparatus. The buchu is less irritating to the stomach than the pareira brava, but cannot alone remove the morbid condition of the mucous membrane, on which the discharge depends. It may, occasionally, be employed with benefit, when combined with other remedies. The ground laurel or *epigea repens*, may sometimes answer a good purpose. The use of *uva ursi* is frequently followed by the best effects. Dr. Gross has found it particularly serviceable in cases attended with excessive morbid sensibility of the neck of the bladder. He thinks it may be advantageously combined with buchu; and, in the class of cases just mentioned, with carbonate of soda or potash.

These several articles are usually employed with better success in combination than alone. The combination to which Dr. Gross is most partial is that of buchu, uva ursi, and cubebs, in the form of tincture, given several times a day, in conjunction with a small quantity of bicarbonate of soda. In cases of cystorrhea, requiring a tonic, the muriated tincture of iron—which evidently exerts a direct influence upon the urinary organs—is called for. Colchicum is indicated when the patient is of the rheumatic or gouty diathesis. Our author has seen happy results follow the use of benzoic acid, when every thing else had failed. He generally exhibits it in union with copaiba, and has known it to act like a charm.

Valuable therapeutic indications are drawn from the condition of the urine, which should always be subjected to analysis. Thus, if this fluid be found acid, the carbonated alkalis will be serviceable. If, on the contrary, the urine be alkaline, the mineral acids are indicated. Anodynes are indispensable, and counter-irritation in the form of a seton, or an issue applied to the supra-pubic region or perineum, is beneficial in the treatment of cystorrhea. The tendency of cantharides to affect the neck of the bladder renders the use of blisters questionable.

Cystorrhea has been much treated within the past few years, by irrigation of the bladder. It seems particularly adapted to those cases which are attended with atony of the muscular fibres. Various medicated injections are sometimes introduced into the bladder for the purpose of acting directly upon the inflamed surface.

Our author has used cauterization—which is so highly recommended by Civiale—in several instances, but without any good results.

When all other remedies have proved inefficient, Mr. Guthrie, of London, recommends that an incision be made into the neck of the bladder, similar to that made in the lateral operation for stone, for the purpose of allowing the mucous secretion to escape as rapidly as it takes place, and, thereby, insure comparative repose to the organ. The principle of the operation is identical with that performed for anal fissures, and fistula. We guard on the one hand against the too speedy closure of the wound, and have a care, on the other, lest it become fistulous. Dr. Parker, of New York, was the first to perform this operation. The case terminated fatally, not, however, until after the expiration of a month, during the greater part of which period, the condition of the patient was greatly improved, and indeed gave rise, at one time, to hopes of his recovery. The mucous surface of the bladder was found studded with numerous miliary tubercles; the muscular coat was considerably hypertrophied. The right kidney was filled with softened tubercular matter. "Both lungs contained tubercles at their summits, and in the left there was a small cavity." Dr. Gross was present at the operation, and says that he should not hesitate to resort to it whenever a favorable opportunity offered. He regards it as "particularly applicable to that form of cystorrhea in which there is marked hypertrophy of the prostate gland, and muscular coat of the bladder."

*Bar-like ridge* of the neck of the bladder, is a peculiar form of hypertrophy sometimes met with, particularly in old subjects. It is the result of causes which produce obstruction to the evacuation of the urine, and consequent retention of this fluid in the bladder. The statement of the causes of this disease is suggestive of its symptoms, which, it need hardly be said, are the same as those which indicate the existence of hypertrophy of the neighboring structures, with mechanical obstruction to the flow of urine. This circumstance gives rise to the difficulty which is experienced in the diagnosis of the lesion. Nearly always coexisting either as cause or effect with stricture of the urethra, chronic enlargement of the prostate gland, general hypertrophy of the bladder, all of which present the phenomena of cystorrhea, the rational signs of this affection are comparatively of little assistance in the diagnosis. The more valuable diagnostic symptoms are furnished by physical exploration, performed with the finger and catheter. If the catheter do not meet with an obstacle in the urethra, if there be no stone and no enlargement of the prostate, the instrument will pass as low down as the orifice of the bladder, where, if there be this ridge, or artificial dam, it will either be completely arrested, or advance with difficulty. "If the finger be now introduced into the rectum, and carried up as high as possible, the point may, if the prostate is nearly of the natural bulk, be hooked round its posterior extremity, and be thus brought directly opposite the bar, and consequently, between it and the back of the instrument."

The treatment is rather palliative than curative. The means recommended in cystorrhea, which forms its most prominent symptom, are also adapted to this disease. The catheter is in constant requisition, being used at regular intervals, or retained in the organ permanently, or removed occasionally for purposes of cleanliness. Scarification of the bar has been proposed by Mr. Guthrie. When the disease proves intractable, our author coincides with Mr. Guthrie in the opinion, that we should afford the patient the benefit of an operation similar to that which is practised for the removal of stone. This procedure would allow of the involuntary escape of the urine and mucus, and if the wound were kept open a sufficient length of time, a new and more healthy action would be almost sure to follow.

*Sacculation of the Bladder*—a striking representation of which is furnished in an original sketch by our author—is the subject of the remaining section of this chapter.

Of this affection we have only room to remark, that Dr. Gross regards it as much more frequent than is generally supposed—that it is the result of mechanical obstruction, situated either in the urethra, or at the neck of the bladder, giving rise to difficulty in voiding the urine; this produces hypertrophy of the muscular tunic, the fibres of which gradually separate from each other, thereby removing, in certain situations, the resistance of this tunic, and allowing the mucous membrane—which is pressed upon on every side by the distended bladder—readily to enter the interstices between the fibres; and, the exciting cause continuing, gradually to project beyond the level of the peritoneal surface. The only symptom of encysted bladder which is possessed of diagnostic value is that of a movable, elastic, circumscribed and fluctuating tumor situated in the hypogastric region. The diagnosis is rendered much more certain if the tumor rises and falls with the quantity of urine contained in the bladder, and should the swelling disappear under the catheter "which may sometimes, by a happy hit, be passed into the abnormal pouch."

The prognosis of this disease is highly unfavorable—the treatment wholly palliative.

We come now to a very interesting portion of the work, that devoted to *Nervous affections.* Irritability, Neuralgia, and Paralysis, are the three nervous affections of the bladder treated of by our author. The first of these diseases is often one of the most difficult of cure with which the practitioner meets. Frequent micturition is its most prominent symptom. This is generally accompanied with tenesmus, a sense of scalding in the urethra, more or less straining, and pain at the neck of the bladder. Occurring at any period of life, and in all temperaments, our author has most frequently met with it in children, and in persons about the age of puberty, and in individuals who are naturally of a nervous, irritable disposition, or prone to attacks of gout and rheumatism. The disease is particularly intractable in scrofulous and weakly habits. Children are subject to a variety of this affection, known generally under the name of incontinence of urine. Those portions of the urinary apparatus which are distinguished for their sensibility both in health and disease—the prostatic portion of the urethra, and neck of the bladder—are the localities to which the disease is most partial. The urine may or may not be altered in its quantity and quality. Generally, it is high colored, loaded with a whitish or grayish mucus, and acid. The stream of water may be natural, spiral, forked, or twisted, strong or feeble, full, or small, discharged in jets, or voided in drops. In most cases of this affection occurring in young men, there is a tendency to erections and seminal emissions. When irritability of the bladder is confirmed and exists in an aggravated form, the general health is gradually impaired; the mind becomes gloomy and despondent; flatulence, colicky pains, constipation, acid eructations, and loss of appetite, combine to harrass and wear away the patient.

Irritability of the bladder depends upon a variety of causes, general and local. All diseases of the urinary apparatus are frequent and well known causes of this disease. One of the most familiar and common causes is gonorrhea. An altered state of the urine is another fruitful source of this malady. Over-doses of diuretics act as irritants of the bladder. The effects of cantharides are well known. Our author has seen nitrate of potash produce intense irritability of the bladder. We have ourselves known a similar result in several cases of rheumatism in which we exhibited this remedy. All drinks, fruits, and vegetables which have a special tendency to develop acid in the urine, with a morbid sensibility of the mucous membrane, may induce this condition of things. Venereal excesses, derangement of the digestive apparatus, lesion of the brain and spinal cord, disease of the alimentary canal and pelvic viscera, disorder of the genital organs, exposure to heat and cold, and general debility, are all exciting causes of this affection.

The idiopathic form of the disease never of itself proving fatal, but little opportunity has been afforded for ascertaining its pathology. So far as we know, or, more strictly speaking, so far as we are justified in concluding, the complaint consists in an exaltation of the nervous sensibility of the mucous membrane. It not unfrequently happens that the irritability is purely sympathetic, depending upon lesion of some neighboring organ, and, in some instances, of a remote organ.

Of course when the disease is produced by a local cause, the anatomical changes are more distinct, and consist of "evidences of inflammation or congestion of the lining membrane, and hypertrophy of the muscular fibres, with alteration of the secretions, and, in some instances, slight deposits of lymph."

The prognosis is entirely dependent upon the nature of the cause, The idiopathic form of the complaint, though obstinate, is, generally, amenable to treatment. When occurring in scrofulous and feeble constitutions, the disease is always intractable, and sometimes irremediable. As may be supposed, when the disease is referable to local causes, and these are of a curable nature, speedy relief may be safely promised. Under opposite circumstances, we anticipate different results.

A knowledge of the cause, so essential indeed in the treatment of even the simplest disorder, is of the first importance in the treatment of this affection. The cause of the disease being known, it is an easy task to distinguish between the cases that are curable and those which are beyond the control of medicine, and to choose from the many remedies possessed by our art for the removal of this affection, those which are adapted to one or another of its forms. Vesical irritability dependent upon stone, or stricture, or hypertrophy of the prostate; vesical irritability dependent upon congestion or inflammation, an altered state of the urine, the improper employment of diuretics, worms, &c., is an affection, the treatment of which is, at once, suggested by its cause. Organic disease of the kidney, ureter, bladder, ovary and rectum, &c., give rise to irritability of the bladder which is incurable. Our author has found the extract of belladonna administered either internally or by external application, to be exceedingly valuable, especially in that form of the complaint which is attended with neuralgic symptoms, or sharp, darting, and shooting pains in the region of the bladder and pelvis. He sometimes uses an ointment of belladonna and strychnine, which is applied to the perineum, sacrum, and lower part of the abdomen. In the irritation of the bladder in young children and hysterical girls, Dr. Gross thinks favorably of the tincture of cantharides. The balsam of copaiba is indicated in those cases which depend upon extension of gonorrheal inflammation, disease of the kidney and vesical catarrh. Dr. Gross places a very different estimate upon buchu and pareira brava from that with which they are spoken of by Brodie, Coulson, and others. "He cannot recall a solitary instance in which they seemed to afford any permanent benefit." His opinion of cubebs is no higher.

Next follows *Neuralgia* of the bladder. From the small space devoted to this subject in other works on the urinary organs, one would be led to conclude that it is an affection seldom met with and but little understood by their authors. So little indeed has this disease been understood, that more than once the operation of lithotomy has been performed for its removal. The late distinguished Dr. Parrish, of Philadelphia, relates a case in which he and the different surgeons in the Pennsylvania Hospital mistook neuralgia of the bladder for vesical calculus. We have ourselves known the same mistake to be made in two cases in this city, and in a case in Tennessee.

The symptoms of this disease, especially in its early stages, are so vague and ill-defined that even the most experienced practitioner will often be in doubt as to their cause; and, on the other hand, when the attacks are severe, they bear the closest resemblance to the paroxysms produced by calculous concretions. The disease is generally paroxysmal in its character. At first the paroxysms recur only at long intervals, and consist merely of a sense of uneasiness in the perineal region, accompanied by a sharp, darting, or tingling pain. Gradually, the attacks become more frequent and more severe, and last from two to six hours, often giving rise to the most intense suffering. During the intermissions, the patient feels comparatively well. The disease often assumes the quotidian type, and generally comes on in the evening or towards morning. Sometimes, however, the attacks have been known to recur twice in the twenty-four hours. However violent or protracted, it is seldom accompanied by fever. Its periodicity, the sharp, darting, and lancinating pains, the *ardor urinæ*, the frequent, urgent, and difficult micturition, and the numbness of the perineum, scrotum, groins, and thighs, are, perhaps, the most important diagnostic signs.

Neuralgia of the bladder occurs, for the most part, in the same class of individuals who are subject to the same malady in other regions of the body; that is to say, in persons of a nervous temperament; and from what we know of this malady, as it occurs in other parts of the body, our author infers that malaria is often a principal, if not the only cause. He attended an elderly gentleman who "was subject to neuralgic attacks of the bladder and right knee, which generally lasted eight or ten days at a time, then disappeared and recurred about once every three months. In early life he had been severely affected with rheumatism, and a short time before the vesical neuralgia came on, he had labored under intermittent fever, which left him with an enlarged and indurated state of the spleen."

The pathology of this disease is unknown. It is singularly intractable. and often resists for weeks, or even months, the best directed treatment; "on the other hand, instances occasionally occur which disappear almost as suddenly as they come on. This is especially the case when the disease has a miasmatic origin, or when it supervenes upon intermittent fever."

The causes of this disease are generally so obscure and inappreciable, and the treatment so entirely regulated by a knowledge of them, that its management is in almost all instances difficult, and often requires the employment of various measures before being finally successful. Our author opposes it with the same remedies which are used for the relief of neuralgia occurring in other parts of the body. "When the disease is connected with an inflammatory state of the system, as it is occasionally found to be, prompt and efficient blood-letting is the remedy upon which, in the commencement, our hopes of success must be mainly placed." Of the utility of local abstractions of blood, our author does not think favorably. He esteems purgatives—of which he thinks the mercurials the best—of much value in this affection, "especially in that variety of it dependent upon a miasmatic origin, constipation of the bowels, or deranged menstrual action." While he thinks that in those cases which are unaccompanied with inflammation or organic disease of the stomach or bowels much advantage may be derived from the judicious use of carbonate of iron, he gives the preference to quinine and arsenic, not only when the neuralgia is seated in the urinary bladder, but also in other parts of the body. When by the lancet, purgatives, and quinine, the intensity of the disease has been mitigated, Dr. Gross thinks arsenic, strychnine, and aconite, in union with opium, the best medicines to eradicate it.

The formula which Dr. Gross has been in the habit of using for many years past, both in this and other forms of neuralgia, and of which he speaks in the highest terms, is the following:

℞. Acid. arseniosi gr. ij.
Strychninæ gr. j.
Ext. aconiti gr. viij.
Pulv. opii gr. v.—M. optim. div. in pil xvj. One pill every six hours.

If they induce nausea, they are to be given less frequently—attention to this point he regards as a matter of paramount importance. They should be omitted at the expiration of every seven or ten days in order to allow the stomach a short recess, when they should be resumed, and taken as before. "Administered with these precautions, arsenic, &c., seldom fail to produce a most favorable impression, and are often, of themselves sufficient to effect a cure." Our author regards Fowler's solution of arsenic as much inferior to arsenic in substance. He exhibits narcotics in full and sustained doses, having previously cleared out the bowels and restored the secretions, and alludes to cases in which radical cures were effected by the protracted exhibition of this class of remedies. Emetics are indicated, particularly when there is associate gastric and biliary disorder. The warm bath, fomentations, the application of steam; colchicum in the gouty and rheumatic habit, and injections of acetate of lead, &c., are mentioned favorably. He has seen so little benefit derived from the introduction of bougies into the bladder—as recommended by M. Civiale—that he has entirely abandoned this mode of treatment. He used the actual cautery on one occasion, but without good effects. Our author completes this section of the chapter on nervous diseases by the detail of four cases of vesical neuralgia, which we much regret our limits forbid us to extract.

*Paralysis* of the bladder occupies the remaining section of the chapter, and is treated of at length and in a masterly manner. We have already, however, consumed so much space by our remarks and have so much yet before us which is of even greater interest than any of the many subjects to which we have yet alluded, that we must content ourselves with a mere synopsis of the teachings of our author on this disease.

Paralysis of the bladder is either partial or complete, essential or symptomatic. External injury, inflammation, disease or injury of the cerebro-spinal axis, loss of tone of the general system, over-distention of the muscular fibres, and old age are its principal causes. Whether dependent upon one of these causes or another, the symptoms which attend it are generally unmistakable. Its characteristic symptom is complete retention of urine. The walls of the bladder are distended by the accumulated fluid, and the organ forms a fluctuating ovoidal tumor in the hypogastric region, which ascends sometimes as high as the umbilicus. "The prognosis of vesical paralysis can be correctly estimated only by an attentive consideration of its causes." The success and also the mode of treatment hinges upon the influences by which the disease has been developed and sustained. To draw off the urine, and restore the tone of the muscular fibres of the affected organ, are the two important indications to be fulfilled in every case. The use of the catheter is sufficient for the first. It is advisable, in some cases, to allow the catheter to remain—the water being permitted to flow off every hour or two—in others, again, it should be introduced only when necessary. The former practice is particularly useful when there is a frequent desire to urinate, attended with pain and spasm of the neck of the bladder. The sudden removal of the stimulus of distention may induce severe depression. Dr. G. has known instances in which it has proved fatal. His practice, when the accumulation is very great, is not only to allow a small quantity of urine to remain, but to support the parts precisely as after tapping in ascites, &c.

To impart tone to the bladder, or, in other words, to meet the second indication, will require a very varied treatment, governed, as we have remarked, by the cause of the disease. The use of emetics and cathartics is nearly always followed by good results, especially where the paralysis occurs coetaneously with disorder of the digestive organs and torpor of the general system. The ground being properly prepared, we may resort, with much confidence, to a class of remedies—at the head of which we place strychnine, arnica, and cantharides—which are calculated to produce a direct impression upon the nervous system, if not upon the bladder itself. They are indicated in the inflammatory form of the affection. Our author thinks highly of arnica in that form of paralysis which occurs as one of the *sequelæ* of masturbation, typhoid, and other fevers. Its virtues are due to its stimulating qualities, and it is, consequently, applicable to cases which are the result of general debility. Dr. Gross has not had much experience in the employment of the ergot of rye, but has never seen it produce good effects. The inflammatory form of the disease is to be met by antiphlogistics. Counter-irritation he regards as a useful auxiliary, and that form, excited by a succession of blisters, will be likely to prove serviceable in every species of paralysis except that just mentioned. The cold douche is a potent remedy. Dr. Gross has seen excellent results ensue from injections into the rectum with strychnine. He thinks it not improbable that this and other analogous remedies might be beneficially introduced, in weak solutions, into the bladder.

*Heterologous formations* of the bladder—embracing scirrhus, encephaloid, tubercular disease, with an allusion to colloid, and melanosis—form the subject of chapter sixth.

Our author reports an exceedingly interesting case of scirrhus of the bladder which occurred in his own practice, and another, for the particulars of which he is indebted to Dr. Church, of the New York Hospital. In the section devoted to encephaloid, he details eight cases of this disease obtained from different sources.

The records of medical science contain no well-authenticated instance of either colloid or melanosis of the bladder.

Tubercular disease of the bladder is occasionally met with. Dr. Gross alludes to a single case furnished by his own practice. Since the publication of his work, one other case has fallen under his observation, reference to which has been made elsewhere. It need hardly be remarked that tubercular disease of the bladder reveals itself by no pathognomonic or even characteristic symptoms. When the deposit exists in a crude state, the symptoms have been known to simulate those of stone. Dupuytren once committed the error of cutting a little boy when he was the subject of tubercles merely.

Polypous, fungous, erectile, and other morbid growths of the bladder, form the subjects of chapter seventh.

Dr. Gross makes mention of a number of cases of this class of affections, occurring in the writings of others. He has seen none himself.

The exciting causes and diagnostic characters of these morbid growths are unknown. Internal remedies exert no influence in arresting or modifying their growth or development. "Whenever, in any manner, their real character can be ascertained, the bladder should be laid open, as in the common operation of cystotomy, and the morbid growth removed with a pair of probe-pointed scissors, curved on the flat." The disease being rarely, if ever, of a malignant character, and, consequently, not liable to reproduction, success would probably attend the procedure, provided the subsequent inflammation, which would generally be considerable, could be controlled.

Chapters eight, nine, ten, eleven, and twelve, treat of *worms in the bladder, serous cysts, and hydatids, fetal remains, hair, and air in the bladder.*

Next follows, in chapter thirteenth, *Hemorrhage* of the bladder. This affection is idiopathic and traumatic. The first is infrequent, and occurs in anemic conditions of the system, as a consequence of typhoid fever, small-pox, &c.

The pathology of the disease varies with its cause. The treatment is conducted with due regard to the nature of the exciting cause. It is both general and local. It sometimes happens that the blood, which is poured out in vesical hemorrhage, is not dissolved by the urine, and conducted off by the natural channel, but coagulates, and accumulating, distends the bladder. A hard, firm tumor above the pubes, a complete retention of urine, attended by extreme distress, &c., are the symptoms. Should the simpler means—among which we may enumerate the introduction of the catheter, and injections of tepid water and acetic acid—fail, and the symptoms are so urgent as not to allow further delay, resort must be had to the operation of opening the bladder. When practicable, the lateral method should always be preferred, both on account of the facility of its execution, and the more dependent situation of the aperture.

*Retention of Urine* forms the subject of chapter fourteenth. The symptoms of this disease are usually characteristic. Its causes and progress are sufficiently well known, but notwithstanding, numerous cases are recorded, in which egregious mistakes were made as to its diagnosis. Dropsy of the peritoneal cavity is the affection with which it is most liable to be confounded. The existence of a hard, pyriform, circumscribed, fluctuating tumor, tender on pressure, fixed, corresponding with the mesian line, and steadily increasing in size, with the general and other local symptoms, are sufficient to enable the practitioner to distinguish this from ascites.

The treatment of retention of urine, is by the catheter. The same precaution which was suggested when speaking of the use of the catheter in paralysis of the bladder, is to be observed in the present instance. The five principal causes of retention of urine are, mechanical obstruction, paralysis, spasm, inflammation, and pelvic tumors—the study of these, with the treatment adapted to each, consumes the remaining portion of this section; and though very instructive, we must hasten on.

Next follows some very practical and judicious remarks upon catheterism, and puncture of the bladder. Our author mentions several very interesting cases in which he thought, at one time, that he would be obliged to resort to the latter operation, but succeeded in overcoming the difficulty by the use of general means and the catheter. He describes the several methods in vogue of puncturing the bladder, and alludes to a singular instance in which the puncture—made in the supra-pubic region—after having been perfectly healed, re-opened after a lapse of fourteen years.

*Incontinence of Urine, and Hernia of the Bladder,* are the remaining subjects until we reach Urinary deposits, and Stone in the bladder, which will occupy the principal part of our next article.

Incontinence of urine may be complete or partial, permanent or temporary. While it may depend upon a great variety of causes, the most prominent are referable to external injury, or to inflammation, spasm, paralysis, or morbid sensibility of the bladder, or of this organ and the urethra.

The symptoms of this affection require no description. The treatment is regulated by the cause. When the disease is irremediable, it only remains for the poor patient to wear a urinal, two cuts of which are given.

*Hernia of the Bladder,* though uncommon, is of great practical interest. The vaginal region is the most common seat of the protusion. The inguinal and femoral regions are also subject to it. When occurring in the latter situations, the treatment does not differ from that of intestinal hernia. Vaginal cystocele is treated by the pessary, cold, astringent injections into the bladder, the frequent use of the catheter, and rest in the recumbent posture.

The diagnosis of cystocele is given by our author at length, and with great clearness. Our limits, however, forbid us delaying longer upon this portion of the work.

